# Novel techniques and developments in migraine research

**DOI:** 10.1186/1129-2377-14-S1-O6

**Published:** 2013-02-21

**Authors:** Simon Akerman

**Affiliations:** 1 University of California San Francisco, USA

## 

Advancing our understanding of the pathophysiology of migraine is one of the key ways that researchers can help provide answers to the many questions that migraineurs have. More importantly it is the only way that tailored therapeutics can be developed that can provide relief to all sufferers, not just those that are lucky enough to respond to existing therapies. It is therefore crucial that we in headache research continue in our efforts to find these answers, through continued development of our methods and providing open-minded thinking to embrace new techniques and ideas into our field. There is much important research in migraine being conducted throughout the world at this time, research that will hopefully help us to find some of these answers. It is the aim and focus of this teaching session to try to introduce the audience to some of the more recent findings and developments in migraine research, and some of the novel approaches researchers are trying to take to understand the dynamics of the migraine pain experience, from a fully translational point of view.

Dr. Akerman will provide an introduction to the topics that will be highlighted and briefly discuss our current understanding of the pathophysiology of migraine and how this may drive migrainous symptoms. It is well known that migraine involves activation of the trigeminovascular system, but it is what and how other areas of the brain and periphery may drive this activation in patients to impact migraine that still requires further investigation. These areas may include the peripheral meningeal nociceptors, the brainstem and the diencephalon, but also interaction of these areas with the cortex, and the respective importance of both the neuronal and vascular components. We know that thalamocortical function is very important in the processing of nociceptive information. Altered cortical function has long been thought to be at the root of migraine aura, and maybe even trigeminovascular activation. Dr. Holland will use preclinical data to discuss how alteration of normal cortical function can result in symptoms such as migraine aura, but also how uncoupling of the normal neurovascular relationship in the cortex, thought to drive aura, can also impact other symptomatic aspects of migraine that results in migraine as a global brain disorder. Dr. Sprenger will discuss this issue of cortical function from a clinical perspective. He will try to address how imaging is helping us understand the role of the cortex in migraine, and how migraineurs may present cortical function that is different from the non-migrainous brain.

Another very important issue for the headache researcher is in actually understanding the dynamics of the pain experienced by the migraineur. Other forms of pain research, away from the head, have for a long time relied on animal behavioural models to observe nociceptive behaviours as a marker of pain. These models are used to study, preclinically, the different dynamics of pain that may be experienced in the clinic and also to test pharmacological agents for therapeutic efficacy. It is only recently that headache research has begun to embrace these techniques. Dr. Romero will discuss some of the methods and techniques that can be used to assess pain in the head and facial region in migraine. She will address the importance of measuring spontaneous (non-evoked) nociceptive behaviours in animals, something which the migraine patient complains of mostly, and how one can do this. Also, as we have seen in our understanding of migraine pathophysiology and the role of sensitisation, how this physiology can be interpreted in the measurement of evoked behaviours in animals. Finally, for a clinician, understanding the pain experienced by their patients is of the utmost importance in diagnosing and treating them, as well as aiding us in understanding the pathophysiology. Therefore, having the tools available to understand this is crucial. Dr. Geber, using this clinical approach, and his skills and experience in allodynia and sensory testing in experimental pain models and pain patients, will describe how these techniques can be used in patients to understand the quality of the pain symptoms related to the headache and facial region. Also, how they can be used experimentally as a research tool to aid us in understanding more about the pathophysiology of migraine and other head pain disorders.

